# Supplementary material for: Participation in community-based health care interventions (CBHIs) and its association with hypertension awareness, control and treatment in Indonesia
Source: PLoS One. 2020 Dec 28;15(12):e0244333. doi: 10.1371/journal.pone.0244333 (PMC7769427; doi:10.1371/journal.pone.0244333)
Supplement: S1 Table — (DOCX) [file pone.0244333.s001.docx]

**Supplementary Table 1** Logistic regression results of participation in community-based health interventions (CBHIs) for non-communicable diseases (NCDs) and other determinants of awareness, treatment, and control among respondents with hypertension as well as control among treated respondents in Indonesia.

|  | **Awareness** | | | **Treatment** | | | **Control (All)** | | | **Control (treated)** | | |
| --- | --- | --- | --- | --- | --- | --- | --- | --- | --- | --- | --- | --- |
|  | **OR** | **95% CI** | **99% CI** | **OR** | **95% CI** | **99% CI** | **OR** | **95% CI** | **99% CI** | **OR** | **95% CI** | **99% CI** |
| Participation in CBHI for NCDs | 1.50‡ | 1.19, 1.89 | 1.11, 2.04 | 2.18‡ | 1.61, 2.95 | 1.46, 3.25 | 1.18 | 0.84, 1.68 | 0.75, 1.87 | 1.80 | 0.92, 3.52 | 0.75, 4.34 |
| Urban | 1.08 | 0.96, 1.22 | 0.92, 1.27 | 1.27* | 1.02, 1.58 | 0.95, 1.70 | 0.93 | 0.79, 1.09 | 0.76, 1.15 | 1.33 | 0.87, 2.02 | 0.76, 2.31 |
| *Age group (reference: 18-39 years old)* |  |  |  |  |  |  |  |  |  |  |  |  |
| Middle-aged (40-59 years old) | 1.17* | 1.03, 1.33 | 0.99, 1.39 | 2.79‡ | 2.09, 3.73 | 1.90, 4.09 | 0.52‡ | 0.44, 0.60 | 0.42, 0.63 | 1.07 | 0.67, 1.69 | 0.58, 1.95 |
| Older-aged (≥60 years old) | 1.28† | 1.08, 1.51 | 1.03, 1.59 | 4.31‡ | 3.10, 5.98 | 2.79, 6.64 | 0.27‡ | 0.21, 0.34 | 0.19, 0.37 | 1.25 | 0.72, 2.19 | 0.60, 2.61 |
| Female | 2.17‡ | 1.93, 2.44 | 1.86,2.53 | 1.77‡ | 1.44, 2.18 | 1.35, 2.32 | 1.15‡ | 1.30, 1.76 | 1.24, 1.84 | 1.44* | 1.01, 2.05 | 0.90, 2.30 |
| Javanese | 0.85* | 0.75, 0.96 | 0.72,1.004 | 0.79* | 0.63, 0.98 | 0.59, 1.04 | 0.78† | 0.66, 0.91 | 0.63, 0.96 | 0.67 | 0.44, 1.02 | 0.38, 1.16 |
| *Marital status, reference: single* |  |  |  |  |  |  |  |  |  |  |  |  |
| Married | 1.31‡ | 1.13, 1.53 | 1.07, 1.60 | 1.22 | 0.94, 1.60 | 0.86, 1.73 | 1.33† | 1.08, 1.63 | 1.01, 1.74 | 1.59 | 0.95, 2.68 | 0.80, 3.16 |
| Separated/widowed | 1.12 | 0.78, 1.60 | 0.69,1.80 | 1.11 | 0.57, 2.14 | 0.47, 2.62 | 0.92 | 0.56, 1.51 | 0.48, 1.76 | 1.02 | 0.18, 5.59 | 0.11, 9.54 |
| *Education, reference: primary school or less* |  |  |  |  |  |  |  |  |  |  |  |  |
| High school | 1.14* | 1.01, 1.30 | 0.96, 1.35 | 1.15 | 0.91, 1.44 | 0.85, 1.55 | 1.26† | 1.06, 1.49 | 1.01, 1.57 | 1.45 | 0.92, 2.28 | 0.80, 2.62 |
| College or higher | 1.31† | 1.06, 1.61 | 0.96, 1.35 | 1.44* | 1.04, 1.99 | 0.94, 2.21 | 1.29 | 0.99, 1.68 | 0.91, 0.82 | 1.73 | 0.93, 3.24 | 0.76, 3.94 |
| *Wealth, reference: poorest quintile (1^st^)* |  |  |  |  |  |  |  |  |  |  |  |  |
| 2^nd^ | 1.07 | 0.89, 1.29 | 0.84, 1.36 | 1.48* | 1.02, 2.15 | 0.90, 2.42 | 1.13 | 0.89, 1.44 | 0.82, 1.55 | 3.56† | 1.51, 8.38 | 1.15, 10.98 |
| 3^rd^ | 1.21* | 1.01, 1.46 | 0.96,1.54 | 1.72† | 1.19, 2.48 | 1.06, 2.78 | 1.16 | 0.91, 1.48 | 0.84, 1.56 | 3.72† | 1.60, 8.62 | 1.23, 11.23 |
| 4^th^ | 1.17 | 0.98, 1.41 | 0.92, 1.49 | 1.81‡ | 1.26, 2.60 | 1.12, 2.92 | 1.14 | 0.90, 1.45 | 0.84, 1.56 | 3.04* | 1.28, 7.17 | 0.98, 9.40 |
| Wealthiest quintile (5^th^) | 1.18 | 0.98, 1.43 | 0.93,1.51 | 2.50‡ | 1.75, 3.56 | 1.57, 3.98 | 1.11 | 0.87, 1.43 | 0.80, 1.54 | 4.07‡ | 1.72, 9.62 | 1.31, 12.60 |
| Health insurance | 1.28‡ | 1.14, 1.44 | 1.10, 1.49 | 1.26* | 1.03, 1.55 | 0.96, 1.66 | 1.08 | 0.93, 1.25 | 0.89, 1.31 | 1.63* | 1.10, 2.41 | 0.97, 2.73 |
| *Geographical areas, reference: Java and Bali* |  |  |  |  |  |  |  |  |  |  |  |  |
| Sumatra | 1.02 | 0.89, 1.18 | 0.85, 1.23 | 0.93 | 0.72, 1.19 | 0.67, 1.29 | 1.14 | 0.96, 1.36 | 0.90, 1.44 | 1.18 | 0.76, 1.82 | 0.66, 2.09 |
| Kalimantan | 1.34† | 1.07, 1.69 | 0.99, 1.81 | 1.70† | 1.19, 2.43 | 1.06, 2.71 | 0.88 | 0.65, 1.19 | 0.59, 1.31 | 0.74 | 0.34, 1.62 | 0.26, 2.08 |
| Sulawesi | 1.02 | 0.78, 1.34 | 0.72,1.45 | 0.48* | 0.26, 0.85 | 0.22, 1.02 | 1.37 | 1.00, 1.88 | 0.90, 2.08 | 0.60 | 0.20, 1.76 | 0.14, 2.46 |
| Papua and other islands | 0.49 | 0.38, 0.64 | 0.35, 0.69 | 0.58* | 0.36, 0.94 | 0.31, 1.10 | 0.55* | 0.38, 0.79 | 0.33, 0.89 | 0.77 | 0.35, 1.70 | 0.27, 2.18 |
| Intercept | 0.27‡ | 0.21, 0.34 | 0.19, 0.37 | 0.01‡ | 0.005, 0.01 | 0.005, 0.01 | 0.18‡ | 0.13, 0.25 | 0.12, 0.28 | 0.002 ‡ | 0.001, 0.006 | 0.001, 0.008 |

**Notes:** OR=Odds Ratio; CI=Confidence Intervals; Sig.: *significant at 5% or less; †significant at 1% or less; ‡ significant at 0.1% or less.
