# Supplementary material for: Participation in community-based health care interventions (CBHIs) and its association with hypertension awareness, control and treatment in Indonesia
Source: PLoS One. 2020 Dec 28;15(12):e0244333. doi: 10.1371/journal.pone.0244333 (PMC7769427; doi:10.1371/journal.pone.0244333)
Supplement: S2 Table — (DOCX) [file pone.0244333.s002.docx]

**Supplementary Table 2** Logistic regression results of participation in community-based health interventions (CBHIs) for non-communicable diseases (NCDs) and other determinants of awareness, treatment, and control among respondents with hypertension as well as control among treated respondents in urban Indonesia.

|  | **Awareness** | | | **Treatment** | | | **Control (All)** | | | **Control (treated)** | | |
| --- | --- | --- | --- | --- | --- | --- | --- | --- | --- | --- | --- | --- |
|  | **OR** | **95% CI** | **99% CI** | **OR** | **95% CI** | **99% CI** | **OR** | **95% CI** | **99% CI** | **OR** | **95% CI** | **99% CI** |
| Participation in CBHI for NCDs | 1.38* | 1.04, 1.83 | 0.95, 1.01 | 2.14‡ | 1.53, 3.00 | 1.38, 3.33 | 1.03 | 0.68, 1.55 | 0.60, 1.77 | 1.57 | 0.80, 3.07 | 0.65, 3.79 |
| *Age group (reference: 18-39 years old)* |  |  |  |  |  |  |  |  |  |  |  |  |
| Middle-aged (40-59 years old) | 1.24† | 1.05, 1.45 | 1.01, 1.53 | 4.04‡ | 2.77, 5.87 | 2.47, 6.60 | 0.48‡ | 0.40, 0.58 | 0.37, 0.62 | 1.73 | 0.97, 3.10 | 0.80, 3.72 |
| Older-aged (≥60 years old) | 1.44‡ | 1.17, 1.77 | 1.09, 1.89 | 5.88‡ | 3.92, 8.81 | 3.46, 10.00 | 0.29‡ | 0.21, 0.39 | 0.19, 0.43 | 2.30* | 1.20, 4.41 | 0.98, 5.42 |
| Female | 2.32‡ | 2.01, 2.68 | 1.92, 2.81 | 1.84‡ | 1.44, 2.35 | 1.33, 2.54 | 1.57‡ | 1.30, 1.90 | 1.23, 2.02 | 1.31 | 0.85, 2.03 | 0.74, 2.32 |
| Javanese | 0.83* | 0.72, 0.97 | 0.68, 1.01 | 0.85 | 0.66, 1.09 | 0.62, 1.17 | 0.78* | 0.64, 0.95 | 0.61, 1.01 | 0.80 | 0.50, 1.28 | 0.43, 1.49 |
| *Marital status, reference: single* |  |  |  |  |  |  |  |  |  |  |  |  |
| Married | 1.33† | 1.11, 1.60 | 1.05, 1.69 | 1.05 | 0.77, 1.43 | 0.70, 1.57 | 1.31* | 1.02, 1.67 | 0.95, 1.81 | 1.56 | 0.86, 2.85 | 0.71, 3.44 |
| Separated/widowed | 0.97 | 0.64, 1.49 | 0.56, 1.70 | 1.04 | 0.52, 2.06 | 0.42, 2.55 | 0.76 | 0.42, 1.36 | 0.35, 1.64 | 0.10* | 0.01, 0.81 | 0.01, 1.55 |
| *Education, reference: primary school or less* |  |  |  |  |  |  |  |  |  |  |  |  |
| High school | 1.14 | 0.97, 1.35 | 0.92, 1.42 | 1.06 | 0.81, 1.38 | 0.75, 1.50 | 1.24 | 1.01, 1.54 | 0.94, 1.65 | 1.45 | 0.84, 2.48 | 0.71, 2.94 |
| College or higher | 1.36* | 1.06, 1.73 | 0.98, 1.87 | 1.39 | 0.95, 2.04 | 0.85, 2.30 | 1.36 | 1.00, 1.87 | 0.90, 2.06 | 2.09* | 1.004, 4.38 | 0.79, 5.52 |
| *Wealth, reference: poorest quintile (1^st^)* |  |  |  |  |  |  |  |  |  |  |  |  |
| 2^nd^ | 1.06 | 0.83, 1.35 | 0.77, 1.46 | 1.56 | 0.96, 2.51 | 0.83, 2.92 | 0.98 | 0.71, 1.35 | 0.64, 1.49 | 2.92 | 0.90, 9.50 | 0.62, 13.77 |
| 3^rd^ | 1.02 | 0.80, 1.03 | 0.74, 1.40 | 1.80* | 1.13, 2.51 | 0.98, 3.30 | 0.82 | 0.60, 1.12 | 0.54, 1.23 | 3.76* | 1.21, 11.75 | 0.84, 16.73 |
| 4^th^ | 1.00 | 0.79, 1.27 | 0.74, 1.36 | 1.96† | 1.24, 3.11 | 1.07, 3.59 | 0.94 | 0.69, 1.26 | 0.63, 1.38 | 3.45* | 1.09, 10.87 | 0.76, 15.59 |
| Wealthiest quintile (5^th^) | 1.09 | 0.86, 1.37 | 0.80, 1.48 | 2.75‡ | 1.75, 4.31 | 1.52, 4.97 | 0.98 | 0.72, 1.34 | 0.66, 1.47 | 4.59† | 1.47, 14.39 | 1.02, 20.60 |
| Health insurance | 1.25† | 1.08, 1.44 | 1.03, 1.51 | 1.41† | 1.10, 1.80 | 1.02, 1.95 | 1.20* | 1.00, 1.44 | 0.94, 1.53 | 1.84† | 1.15, 2.95 | 0.99, 3.42 |
| *Geographical areas, reference: Java and Bali* |  |  |  |  |  |  |  |  |  |  |  |  |
| Sumatra | 1.09 | 0.90, 1.32 | 0.85, 1.40 | 1.09 | 0.81, 1.46 | 0.74, 1.60 | 1.55‡ | 1.24, 1.94 | 1.16, 2.08 | 1.50 | 0.90, 2.50 | 0.77, 2.94 |
| Kalimantan | 1.36† | 0.98, 1.88 | 0.88, 2.09 | 1.57 | 0.95, 2.62 | 0.81, 3.07 | 0.82 | 0.51, 1.32 | 0.44, 1.53 | 0.85 | 0.30, 2.39 | 0.21, 3.31 |
| Sulawesi | 0.86 | 0.60, 1.24 | 0.53, 1.39 | 0.19 | 0.06, 0.55 | 0.05, 0.76 | 1.28 | 0.82, 2.00 | 0.71, 2.30 | 0.60 | 0.14, 2.46 | 0.09, 3.84 |
| Other islands | 0.45 | 0.32, 0.63 | 0.28, 0.70 | 0.65 | 0.37, 1.15 | 0.31, 1.37 | 0.54* | 0.33, 0.89 | 0.28, 1.04 | 0.88 | 0.34, 2.29 | 0.25, 3.10 |
| Intercept | 0.29‡ | 0.21, 0.40 | 0.19, 0.44 | 0.01‡ | 0.004, 0.01 | 0.003, 0.02 | 0.19‡ | 0.13, 0.28 | 0.11, 0.31 | 0.001‡ | 0.001, 0.005 | 0.0003, 0.007 |

**Notes:** OR=Odds Ratio; CI=Confidence Intervals; Sig.: *significant at 5% or less; †significant at 1% or less; ‡ significant at 0.1% or less.
