# Supplementary material for: Participation in community-based health care interventions (CBHIs) and its association with hypertension awareness, control and treatment in Indonesia
Source: PLoS One. 2020 Dec 28;15(12):e0244333. doi: 10.1371/journal.pone.0244333 (PMC7769427; doi:10.1371/journal.pone.0244333)
Supplement: S3 Table — (DOCX) [file pone.0244333.s003.docx]

**Supplementary Table 3** Logistic regression results of participation in community-based health interventions (CBHIs) for non-communicable diseases (NCDs) and other determinants of awareness, treatment, and control among respondents with hypertension as well as control among treated respondents in rural Indonesia.

|  | **Awareness** | | | **Treatment** | | | **Control (All)** | | | **Control (treated)** | | |
| --- | --- | --- | --- | --- | --- | --- | --- | --- | --- | --- | --- | --- |
|  | **OR** | **95% CI** | **99% CI** | **OR** | **95% CI** | **99% CI** | **OR** | **95% CI** | **99% CI** | **OR** | **95% CI** | **99% CI** |
| Participation in CBHI for NCDs | 1.66* | 1.11, 2.47 | 0.98, 2.80 | 2.14* | 1.11, 4.12 | 0.90, 5.06 | 1.40 | 0.77, 2.54 | 0.63, 3.07 | 2.24 | 0.50, 9.99 | 0.31, 15.98 |
| *Age group (reference: 18-39 years old)* |  |  |  |  |  |  |  |  |  |  |  |  |
| Middle-aged (40-59 years old) | 1.12 | 0.91, 1.38 | 0.85, 1.47 | 1.86† | 1.18, 2.93 | 1.02, 3.38 | 0.58‡ | 0.45, 0.74 | 0.42, 0.60 | 0.59 | 0.28, 1.26 | 0.22, 1.59 |
| Older-aged (≥60 years old) | 1.13 | 0.86, 1.47 | 0.79, 1.60 | 3.07‡ | 1.76, 5.35 | 1.48, 6.36 | 0.25‡ | 0.16, 0.38 | 0.14, 0.43 | 0.51 | 0.17, 1.49 | 0.12, 2.08 |
| Female | 2.01‡ | 1.67, 2.42 | 1.58, 2.56 | 1.65‡ | 1.15, 2.36 | 1.03, 2.64 | 1.44† | 1.13, 1.83 | 1.05, 1.97 | 1.65 | 0.88, 3.11 | 0.72, 3.80 |
| Javanese | 0.86 | 0.70, 1.05 | 0.65, 1.12 | 0.68 | 0.45, 1.03 | 0.40, 1.17 | 0.76 | 0.58, 1.002 | 0.53, 1.09 | 0.46 | 0.20, 1.07 | 0.15, 1.39 |
| *Marital status, reference: single* |  |  |  |  |  |  |  |  |  |  |  |  |
| Married | 1.29* | 1.003, 1.68 | 0.92, 1.82 | 1.63 | 0.96, 2.78 | 0.81, 3.29 | 1.39 | 0.97, 2.01 | 0.86, 2.25 | 1.53 | 0.54, 4.33 | 0.39, 6.00 |
| Separated/widowed | 1.34 | 0.73, 2.43 | 0.61, 2.93 | 1.36 | 0.34, 5.41 | 0.22, 8.35 | 1.20 | 0.55, 2.61 | 0.43, 3.34 | 3.22 | 0.43, 23.68 | 0.23, 44.35 |
| *Education, reference: primary school or less* |  |  |  |  |  |  |  |  |  |  |  |  |
| High school | 1.17 | 0.94, 1.45 | 0.88, 1.55 | 1.29 | 0.84, 1.96 | 0.74, 2.24 | 1.35* | 1.03, 1.78 | 0.94, 1.94 | 1.48 | 0.68, 3.23 | 0.53, 4.12 |
| College or higher | 1.28 | 0.87, 1.89 | 0.77, 2.14 | 1.48 | 0.76, 2.43 | 0.62, 3.52 | 1.14 | 0.69, 1.86 | 0.59, 2.18 | 0.41 | 0.07, 2.44 | 0.04, 4.26 |
| *Wealth, reference: poorest quintile (1^st^)* |  |  |  |  |  |  |  |  |  |  |  |  |
| 2^nd^ | 1.06 | 0.81, 1.39 | 0.75, 1.51 | 1.36 | 0.76, 2.43 | 0.63, 2.92 | 1.23 | 0.86, 1.75 | 0.77, 1.95 | 4.36* | 1.27, 14.91 | 0.86, 21.95 |
| 3^rd^ | 1.43† | 1.09, 1.87 | 1.00, 2.04 | 1.65 | 0.92, 2.94 | 0.77, 3.52 | 1.56* | 1.08, 2.24 | 0.96, 2.51 | 3.78* | 1.03, 13.78 | 0.69, 20.70 |
| 4^th^ | 1.36* | 1.02, 1.80 | 0.94, 1.96 | 1.71 | 0.96, 3.02 | 0.80, 3.62 | 1.33 | 0.92, 1.92 | 0.82, 2.16 | 2.88 | 0.71, 11.53 | 0.46, 17.85 |
| Wealthiest quintile (5^th^) | 1.23 | 0.91, 1.68 | 0.82, 1.85 | 2.19† | 1.21, 3.96 | 1.01, 4.77 | 1.10 | 0.72, 1.68 | 0.63, 1.92 | 2.81 | 0.66, 11.90 | 0.42, 18.74 |
| Health insurance | 1.32† | 1.09, 1.59 | 1.03, 1.69 | 1.08 | 0.74, 1.57 | 0.66, 1.76 | 0.97 | 0.76, 1.24 | 0.71, 1.34 | 1.35 | 0.67, 2.70 | 0.54, 3.36 |
| *Geographical areas, reference: Java and Bali* |  |  |  |  |  |  |  |  |  |  |  |  |
| Sumatra | 0.98 | 0.80, 1.20 | 0.75, 1.28 | 0.76 | 0.50, 1.16 | 0.44, 1.32 | 0.88 | 0.68, 1.15 | 0.63, 1.25 | 0.82 | 0.39, 1.68 | 0.31, 2.12 |
| Kalimantan | 1.33 | 0.97, 1.82 | 0.88, 2.01 | 1.64 | 0.98, 2.72 | 0.84, 3.20 | 0.88 | 0.59, 1.33 | 0.51, 1.51 | 0.52 | 0.16, 1.66 | 0.11, 2.39 |
| Sulawesi | 1.17 | 0.80, 1.72 | 0.71, 1.95 | 0.73 | 0.35, 1.51 | 0.28, 1.90 | 1.45 | 0.92, 2.28 | 0.80, 2.63 | 0.51 | 0.09, 2.69 | 0.05, 4.54 |
| Other islands | 0.53† | 0.36, 0.80 | 0.31, 0.91 | 0.46 | 0.19, 1.13 | 0.14, 1.50 | 0.56* | 0.32, 0.98 | 0.26, 1.17 | 0.62 | 0.15, 2.47 | 0.10, 3.82 |
| Intercept | 0.27‡ | 0.18, 0.41 | 0.16, 0.46 | 0.01‡ | 0.006, 0.03 | 0.004, 0.04 | 0.17‡ | 0.10, 0.28 | 0.08, 0.33 | 0.005‡ | 0.001, 0.03 | 0.0005, 0.05 |

**Notes:** OR=Odds Ratio; CI=Confidence Intervals; Sig.: *significant at 5% or less; †significant at 1% or less; ‡ significant at 0.1% or less.
