# Supplementary material for: Participation in community-based health care interventions (CBHIs) and its association with hypertension awareness, control and treatment in Indonesia
Source: PLoS One. 2020 Dec 28;15(12):e0244333. doi: 10.1371/journal.pone.0244333 (PMC7769427; doi:10.1371/journal.pone.0244333)
Supplement: S4 Table — (DOCX) [file pone.0244333.s004.docx]

**Supplementary Table 4** Poisson regression results of participation in community-based health interventions (CBHIs) for non-communicable diseases (NCDs) and other determinants of awareness, treatment, and control among respondents with hypertension as well as control among treated respondents in Indonesia.

|  | **Awareness** | | **Treatment** | | **Control (All)** | | **Control (treated)** | |
| --- | --- | --- | --- | --- | --- | --- | --- | --- |
|  | **IRR** | **95% CI** | **IRR** | **95% CI** | **IRR** | **95% CI** | **IRR** | **95% CI** |
| Participation in CBHI for NCDs | 1.18‡ | 1.06, 1.30 | 1.79‡ | 1.42, 2.25 | 0.98 | 0.76, 1.27 | 1.30 | 0.74, 2.27 |
| Urban | 1.03 | 0.97, 1.09 | 1.22* | 1.02, 1.44 | 0.98 | 0.88, 1.09 | 1.34 | 0.93, 1.92 |
| *Age group (reference: 18-39 years old)* |  |  |  |  |  |  |  |  |
| Middle-aged (40-59 years old) | 1.06 | 0.99, 1.12 | 2.68‡ | 2.12, 3.38 | 0.58‡ | 0.52, 0.64 | 1.04 | 0.71, 1.53 |
| Older-aged (≥60 years old) | 1.19‡ | 1.10, 1.29 | 4.36‡ | 3.37, 5.64 | 0.36‡ | 0.30, 0.44 | 1.66* | 1.05, 2.61 |
| Female | 1.54‡ | 1.46, 1.63 | 1.72‡ | 1.46, 2.03 | 1.44‡ | 1.30, 1.59 | 1.71‡ | 1.23, 2.36 |
| Javanese | 0.92† | 0.87, 0.97 | 0.84* | 0.71, 0.99 | 0.80‡ | 0.72, 0.89 | 0.65* | 0.46, 0.93 |
| *Marital status, reference: single* |  |  |  |  |  |  |  |  |
| Married | 1.20‡ | 1.11, 1.29 | 1.20 | 0.98, 1.47 | 1.25† | 1.08, 1.44 | 1.57* | 1.01, 2.47 |
| Separated/widowed | 1.09 | 0.92, 1.29 | 1.07 | 0.66, 1.72 | 1.02 | 0.72, 1.43 | 0.98 | 0.30, 3.16 |
| *Education, reference: primary school or less* |  |  |  |  |  |  |  |  |
| High school | 1.09† | 1.03, 1.16 | 1.11 | 0.92, 1.32 | 1.22‡ | 1.08, 1.37 | 1.42 | 0.98, 2.06 |
| College or higher | 1.17‡ | 1.07, 1.29 | 1.47† | 1.14, 1.89 | 1.27† | 1.07, 1.51 | 1.69* | 1.02, 2.80 |
| *Wealth, reference: poorest quintile (1^st^)* |  |  |  |  |  |  |  |  |
| 2^nd^ | 1.04 | 0.95, 1.13 | 1.49† | 1.11, 2.01 | 1.06 | 0.90, 1.25 | 3.24† | 1.50, 6.97 |
| 3^rd^ | 1.06 | 0.98, 1.16 | 1.66‡ | 1.25, 2.21 | 0.99 | 0.84, 1.17 | 3.66‡ | 1.72, 7.76 |
| 4^th^ | 1.09* | 1.005, 1.18 | 1.74‡ | 1.31, 2.31 | 1.08 | 0.92, 1.26 | 3.13† | 1.47, 6.69 |
| Wealthiest quintile (5^th^) | 1.06 | 0.97, 1.16 | 2.21‡ | 1.67, 2.91 | 1.04 | 0.88, 1.23 | 4.27‡ | 2.03, 9.00 |
| Health insurance | 1.12‡ | 1.06, 1.18 | 1.21* | 1.03, 1.42 | 1.06 | 0.96, 1.17 | 1.46* | 1.05, 2.05 |
| *Geographical areas, reference: Java and Bali* |  |  |  |  |  |  |  |  |
| Sumatra | 1.05 | 0.98, 1.12 | 1.001 | 0.82, 1.21 | 1.18† | 1.04, 1.33 | 1.22* | 0.83, 1.78 |
| Kalimantan | 1.15† | 1.03, 1.28 | 1.69‡ | 1.28, 2.23 | 0.85 | 0.68, 1.06 | 1.01 | 0.50, 1.98 |
| Sulawesi | 1.002 | 0.88, 1.14 | 0.58* | 0.35, 0.94 | 1.20 | 0.96, 1.49 | 0.71 | 0.29, 1.75 |
| Other islands | 0.64‡ | 0.56, 0.74 | 0.65* | 0.43, 0.98 | 0.55‡ | 0.42, 0.72 | 0.86 | 0.43, 1.71 |
| Intercept | 0.23‡ | 0.21, 0.26 | 0.009‡ | 0.006, 0.01 | 0.16‡ | 0.13, 0.20 | 0.002‡ | 0.001, 0.005 |

Notes: IRR=Incidence Rate Ratio; CI=Confidence Intervals; Sig.: *significant at 5% or less; †significant at 1% or less; ‡ significant at 0.1% or less.
