# Supplementary material for: Participation in community-based health care interventions (CBHIs) and its association with hypertension awareness, control and treatment in Indonesia
Source: PLoS One. 2020 Dec 28;15(12):e0244333. doi: 10.1371/journal.pone.0244333 (PMC7769427; doi:10.1371/journal.pone.0244333)
Supplement: S5 Table — (DOCX) [file pone.0244333.s005.docx]

**Supplementary Table 5** Poisson regression results of participation in community-based health interventions (CBHIs) for non-communicable diseases (NCDs) and other determinants of awareness, treatment, and control among respondents with hypertension as well as control among treated respondents in urban Indonesia.

|  | **Awareness** | | **Treatment** | | **Control (All)** | | **Control (treated)** | |
| --- | --- | --- | --- | --- | --- | --- | --- | --- |
|  | **IRR** | **95% CI** | **IRR** | **95% CI** | **IRR** | **95% CI** | **IRR** | **95% CI** |
| Participation in CBHI for NCDs | 1.14* | 1.01, 1.28 | 1.78‡ | 1.38, 2.29 | 0.93 | 0.68, 1.27 | 1.28 | 0.70, 2.35 |
| *Age group (reference: 18-39 years old)* |  |  |  |  |  |  |  |  |
| Middle-aged (40-59 years old) | 1.10† | 1.02, 1.19 | 3.27‡ | 2.43, 4.39 | 0.58‡ | 0.50, 0.66 | 1.31 | 0.82, 2.11 |
| Older-aged (≥60 years old) | 1.25‡ | 1.13, 1.38 | 5.15‡ | 3.73, 7.10 | 0.39‡ | 0.31, 0.50 | 2.21† | 1.29, 3.81 |
| Female | 1.60‡ | 1.50, 1.72 | 1.75‡ | 1.44, 2.13 | 1.52‡ | 1.34, 1.74 | 1.72† | 1.18, 2.52 |
| Javanese | 0.93* | 0.86, 0.99 | 0.91 | 0.75, 1.11 | 0.82† | 0.71, 0.94 | 0.80 | 0.54, 1.19 |
| *Marital status, reference: single* |  |  |  |  |  |  |  |  |
| Married | 1.21‡ | 1.10, 1.32 | 1.10 | 0.87, 1.38 | 1.25† | 1.05, 1.50 | 1.65 | 0.98, 2.77 |
| Separated/widowed | 1.06 | 0.86, 1.30 | 1.11 | 0.65, 1.88 | 0.93 | 0.60, 1.45 | 0.46 | 0.06, 3.21 |
| *Education, reference: primary school or less* |  |  |  |  |  |  |  |  |
| High school | 1.10* | 1.02, 1.18 | 1.08 | 0.88, 1.34 | 1.22* | 1.04, 1.42 | 1.50 | 0.95, 2.35 |
| College or higher | 1.18† | 1.06, 1.32 | 1.47† | 1.10, 1.94 | 1.28* | 1.03, 1.58 | 2.04* | 1.16, 3.58 |
| *Wealth, reference: poorest quintile (1^st^)* |  |  |  |  |  |  |  |  |
| 2^nd^ | 1.05 | 0.93, 1.18 | 1.53* | 1.05, 2.24 | 1.04 | 0.83, 1.30 | 3.21* | 1.10, 9.31 |
| 3^rd^ | 1.01 | 0.90, 1.12 | 1.65† | 1.14, 2.37 | 0.90 | 0.72, 1.13 | 4.30† | 1.55, 11.91 |
| 4^th^ | 1.02 | 0.91, 1.14 | 1.81‡ | 1.27, 2.58 | 1.02 | 0.82, 1.26 | 3.80† | 1.37, 10.52 |
| Wealthiest quintile (5^th^) | 1.02 | 0.91, 1.14 | 2.28‡ | 1.62, 3.22 | 1.03 | 0.83, 1.28 | 5.05‡ | 1.85, 13.72 |
| Health insurance | 1.11‡ | 1.04, 1.94 | 1.31† | 1.08, 1.59 | 1.08 | 0.95, 1.23 | 1.44 | 0.96, 2.15 |
| *Geographical areas, reference: Java and Bali* |  |  |  |  |  |  |  |  |
| Sumatra | 1.07 | 0.98, 1.17 | 1.16 | 0.92, 1.46 | 1.35‡ | 1.16, 1.57 | 1.55* | 1.01, 2.40 |
| Kalimantan | 1.12 | 0.96, 1.30 | 1.52* | 1.04, 2.23 | 0.78 | 0.55, 1.10 | 1.12 | 0.46, 2.69 |
| Sulawesi | 0.93 | 0.77, 1.11 | 0.33† | 0.14, 0.76 | 1.17 | 0.85, 1.60 | 0.82 | 0.26, 2.53 |
| Other islands | 0.63‡ | 0.52, 0.76 | 0.76 | 0.47, 1.21 | 0.54† | 0.37, 0.80 | 1.03 | 0.45, 2.34 |
| Intercept | 0.23‡ | 0.20, 0.27 | 0.01‡ | 0.005, 0.01 | 0.15‡ | 0.11, 0.20 | 0.001‡ | 0.004, 0.005 |

Notes: IRR=Incidence Rate Ratio; CI=Confidence Intervals; Sig.: *significant at 5% or less; †significant at 1% or less; ‡ significant at 0.1% or less.
