# Supplementary material for: Participation in community-based health care interventions (CBHIs) and its association with hypertension awareness, control and treatment in Indonesia
Source: PLoS One. 2020 Dec 28;15(12):e0244333. doi: 10.1371/journal.pone.0244333 (PMC7769427; doi:10.1371/journal.pone.0244333)
Supplement: S6 Table — (DOCX) [file pone.0244333.s006.docx]

**Supplementary Table 6** Poisson regression results of participation in community-based health interventions (CBHIs) for non-communicable diseases (NCDs) and other determinants of awareness, treatment, and control among respondents with hypertension as well as control among treated respondents in rural Indonesia.

|  | **Awareness** | | **Treatment** | | **Control (All)** | | **Control (treated)** | |
| --- | --- | --- | --- | --- | --- | --- | --- | --- |
|  | **IRR** | **95% CI** | **IRR** | **95% CI** | **IRR** | **95% CI** | **IRR** | **95% CI** |
| Participation in CBHI for NCDs | 1.25* | 1.03, 1.51 | 1.65 | 0.98, 2.77 | 1.08 | 0.70, 1.66 | 1.20 | 0.50, 9.99 |
| *Age group (reference: 18-39 years old)* |  |  |  |  |  |  |  |  |
| Middle-aged (40-59 years old) | 0.99 | 0.90, 1.10 | 1.91‡ | 1.30, 2.81 | 0.59‡ | 0.49, 0.70 | 0.65 | 0.32, 1.29 |
| Older-aged (≥60 years old) | 1.10 | 0.96, 1.25 | 3.32‡ | 2.15, 5.12 | 0.32‡ | 0.24, 0.44 | 0.82 | 0.35, 1.93 |
| Female | 1.44‡ | 1.32, 1.58 | 1.62‡ | 1.21, 2.18 | 1.31‡ | 1.12, 1.54 | 1.59 | 0.86, 2.93 |
| Javanese | 0.90* | 0.81, 0.99 | 0.69* | 0.50, 0.95 | 0.76† | 0.64, 0.91 | 0.34† | 0.17, 0.71 |
| *Marital status, reference: single* |  |  |  |  |  |  |  |  |
| Married | 1.18† | 1.04, 1.34 | 1.54* | 1.01, 2.34 | 1.25 | 0.97, 1.60 | 1.27 | 0.53, 3.02 |
| Separated/widowed | 1.16 | 0.87, 1.54 | 1.01 | 0.36, 2.82 | 1.18 | 0.68, 2.03 | 2.19 | 0.46, 10.42 |
| *Education, reference: primary school or less* |  |  |  |  |  |  |  |  |
| High school | 1.09 | 0.98, 1.20 | 1.13 | 0.81, 1.58 | 1.24* | 1.04, 1.49 | 1.27 | 0.65, 2.47 |
| College or higher | 1.20† | 1.01, 1.42 | 1.48 | 0.87, 2.50 | 1.26 | 0.93, 1.71 | 0.60 | 0.14, 2.59 |
| *Wealth, reference: poorest quintile (1^st^)* |  |  |  |  |  |  |  |  |
| 2^nd^ | 1.01 | 0.89, 1.16 | 1.36 | 0.85, 2.17 | 1.06 | 0.84, 1.34 | 3.35* | 1.13, 9.88 |
| 3^rd^ | 1.14* | 1.004, 1.30 | 1.68 | 1.06, 2.66 | 1.11 | 0.87, 1.41 | 2.75 | 0.89, 8.42 |
| 4^th^ | 1.20† | 1.05, 1.37 | 1.67 | 1.05, 2.66 | 1.14 | 0.89, 1.45 | 2.51 | 0.78, 8.07 |
| Wealthiest quintile (5^th^) | 1.12 | 0.96, 1.29 | 2.03† | 1.25, 3.27 | 0.99 | 0.75, 1.30 | 3.07 | 0.94, 10.00 |
|  |  |  |  |  |  |  |  |  |
| Health insurance | 1.12† | 1.03, 1.22 | 0.99 | 0.74, 1.33 | 1.03 | 0.88, 1.21 | 1.44 | 0.80, 2.58 |
| *Geographical areas, reference: Java and Bali* |  |  |  |  |  |  |  |  |
| Sumatra | 0.90 | 0.92, 1.13 | 0.74 | 0.50, 0.95 | 0.98 | 0.81, 1.18 | 0.65 | 0.32, 1.33 |
| Kalimantan | 1.16 | 0.99, 1.35 | 1.65* | 0.98, 1.05 | 0.86 | 0.63, 1.16 | 0.61 | 0.21, 1.76 |
| Sulawesi | 1.07 | 0.88, 1.29 | 0.80 | 0.35, 2.52 | 1.18 | 0.86, 1.62 | 0.44 | 0.10, 1.83 |
| Other islands | 0.64‡ | 0.51, 0.80 | 0.45* | 0.19, 0.98 | 0.54† | 0.36, 0.80 | 0.51 | 0.15, 1.71 |
| Intercept | 0.25‡ | 0.20, 0.31 | 0.01‡ | 0.01, 0.02 | 0.18‡ | 0.12, 0.26 | 0.008‡ | 0.001, 0.03 |

Notes: IRR=Incidence Rate Ratio; CI=Confidence Intervals; Sig.: *significant at 5% or less; †significant at 1% or less; ‡ significant at 0.1% or less.
