# Supplementary material for: Participation in community-based health care interventions (CBHIs) and its association with hypertension awareness, control and treatment in Indonesia
Source: PLoS One. 2020 Dec 28;15(12):e0244333. doi: 10.1371/journal.pone.0244333 (PMC7769427; doi:10.1371/journal.pone.0244333)
Supplement: Attachment — Submitted filename: Response for reviewers.docx [file pone.0244333.s007.docx]

Malang, October 23 2020.

To: Dr Geofrey Musinguzi, Academic Editor, PLOS ONE.

Re: Revised Manuscript (ID PONE-D-20-22592)

Dear Dr Musinguzi,

We thank you for your interest in our submission to PLOS ONE and for the opportunity to revise and resubmit our manuscript. We appreciate the extensive comments provided following the peer review, each of which we have considered carefully. In response, we have undertaken a substantial revision of our manuscript in line with the issues and suggestions raised by the three reviewers. For clarity, we have composed a general response for all three reviewers below, followed by a point-by-point response to each reviewer’s comments and suggestions.

We genuinely hope that our manuscript now meets your approval for publication in PLOS ONE.

Yours sincerely,

Dr Sujarwoto

Dr Asri Maharani

**Responses for all three reviewers**

- As suggested by all reviewers, the discussion section has been restructured.
- As suggested by Reviewer 3, the title of this paper has been changed to “Participation in a CBHI and its association with hypertension awareness, control and treatment in Indonesia”
- As suggested by Reviewer 3, we have added gaps in the existing literature on CBHIs and hypertension awareness, treatment and control in LMICs. Some information on settings were moved to the introduction.

**Comments from Reviewer #1 (green colour)**

Thank you for inviting me to review this article. By reporting on population-wide impacts of CBHI programs, where past research has report pilot or initial RCT findings, the current manuscript makes an important and novel contribution to the literature on CBHI for non-communicable diseases in low- and medium-income countries. The manuscript is well written and, aside from a few issues the methodological/statistical, the analysis is robust and well reported.

However, I do have some concerns with the article which I feel must be addressed before I feel that it is suitable for publication. These can largely be split into three categories: [1] issues with the methodology, particularly concerning the time-period over which the data was collected, [2] statistical issues, particularly the lack of control for multiple comparison, [3] structural and content issues with the text, particularly in the discussion.

I have split by comments into major and minor comments which are bullet pointed below. I feel that all these issues can be addressed by the author in some way and once done I believe that the manuscript could be suitable for publication.

**Major Comments:**

• I was a little confused about the time periods that the CBHI program was launched in relation to the data collection period for the IFLS-5. The author states that the CBHI programs were launched in 2015 however the IFLS-5 was collected in 2014. Based on this reading the data will not capture the relevant time-period to analyse the policy. Perhaps there is a history of CBHI which predates the 2015 policy intervention however one might expect that the implementation of the 2015 policy will have had impacts over the last 5 years meaning that the findings of this paper are somewhat out of date. It may just be that the author needs to add more clarity to the setting section of the introduction to explain this issue to the reader.

**Authors’ response**

Many thanks for correcting this. Based on the Non-Communicable Diseases Control and Prevention (P2PTM) Ministry of Health Report 2014, the *Posbindu* program has been implemented as a government program since 2011. The embryo of *Posbindu* is *Posyandu*, which focuses on family planning and has been implemented since 1989. We have changed the year in the revised manuscript. It now reads as 2011 (Ln 123).

**Comment from Reviewer #1**

• I believe that the p-threshold used in the analysis is too lenient. Given that the author has conducted multiple comparisons of a large sample size I believe corrections for multiple comparisons should be applied. This is mediated by the face that CIs are reported however I think if the analysis is going to rely on CIs alone then 99% C/is should also be reported.

**Authors’ response**

We have reported the 99% confidence intervals in Supplementary Tables 1 to 3.

**Comment from Reviewer #1**

• I wondered whether ethnicity and geographic region could be included as IVs for the logistic regression given that Indonesia is a culturally diverse and geographically spread out country. The author picks up on the latter of these issues by reporting that “CBHIs are inequitably distributed across Indonesia” (Ln 134).

**Authors’ response**

We have included ethnicity and geographical area as independent variables in all analyses. We classified ethnicity into Javanese and non-Javanese (reference group) and categorised geographical areas into Java (reference group), Sumatera, Kalimantan, Sulawesi, and other islands (Ln 216-218).

**Comment from Reviewer #1**

• I think the discussion section needs some work. It begins with a summarised repetition of the introduction which I believe is not necessary before addressing three key findings, two of which are not novel and are not related to aims and objectives of this study. I think the discussion should focus more on the CBHI and should open with a section which covers how the analysis addressed the aims and objectives of the study.

**Authors’ response**

We have revised the discussion section. As suggested, we now start the discussion with how we analysed the aim of the study. We then discuss the findings that are related to the aim of the study. We include findings from descriptive analyses as we believe these findings may serve as an introduction for readers before the main findings (Ln 363-452).

**Comment from Reviewer #1**

• The figures are in a low resolution and the text is often too small to read, particularly for figure 1.

**Authors’ response**

We have revised the figures, especially Figure 1, to increase the resolution and the size of the text.

**Comment from Reviewer #1**

**Minor comments:**

• I had some concern about the way the author defined control of hypertension (Ln 168-170) as it does not seem to account for those individuals who may have controlled their hypertension through behaviour change or other treatments. Perhaps this comment is not relevant because CBHI programs in Indonesia may focus specifically on antihypertensive medication. I am not sure, and I am happy to be corrected on this issue as I am not an expert on Indonesian CVD healthcare policy. However, the lack of coverage of behavioural adjustments in article’s definition of control may account in part for the lack of association between CBHI and hypertension control in the analysis where other research has found such associations.

**Authors’ response**

Respondents were considered to have their hypertension under control if they reported that they were on antihypertensive medication and had a mean SBP of < 140 mmHg and DBP of < 90 mmHg. We now mention on Ln 433-435 that “this study did not capture non-pharmacological treatment strategies such as modifications in diet and physical activity, which may have yielded overestimation of the effect of CBHI participation on the reduction of hypertension rates.”

**Comment from Reviewer #1**

• I think there needs to be more justification of the decision to split the analysis by residential area. Clearly, it is an important distinction when it comes to this issue however I feel as though the author has not described why such a decision was made going into the analysis. Currently, this decision feels like it comes out of nowhere on Ln 186. Additionally, it was not clear how rural vs urban was defined.

**Authors’ response**

Justification of the decision to split the analysis by residential area is provided on Ln 237-241. The definition of urban and rural area is based on the Indonesia Spatial Planning Act Number 26/2007, as stated now in Ln 243-249.

**Comment from Reviewer #1**

• Percentage rates for attendance of CBHIs is reported in the discussion but not in the results. I believe it should be reported in the results section.

**Authors’ response**

We now report the percentage rates for attendance of CBHIs in the results (Ln 282).

**Comment from Reviewer #1**

• Sentences on Ln 82-84 beginning with “individuals…” should be reworded as they are a little unclear, particular the second sentence beginning with “Otherwise…”

**Authors’ response**

We have removed the sentence “Individuals who become hypertensive should be identified and treated early, and their elevated blood pressure should be controlled” (Ln 73-74).

**Comment from Reviewer #1**

• I don’t believe that the references for the “low-manpower claim” justify this statement specifically for Indonesia (Ln83-84).

**Authors’ response**

In this sentence, we discuss the case of LMICs in general. In Indonesia, based on the report from the Ministry of Health, the ratio of physicians to population is 4 physicians per 10,000 population which is the second lowest among South East Asian countries. https://databoks.katadata.co.id/datapublish/2020/04/02/rasio-dokter-indonesia-terendah-kedua-di-asia-tenggara

**Comment from Reviewer #1**

• In the abstract and in the methods section (Ln 155) the author refers to participation rates of 93%. Can the author report what this refers to? 93% of whom. Does it mean that of those contacted to take part, 93% agree, or that the retention rate from the IFLS-4 was 93%. I couldn’t find the data in the cited RAND Corp article.

**Authors’ response**

Thank you for correcting us. We have removed the figure of 93% participant rates in the abstract. In the data section, we have added a sentence stating that “the dynasty recontact rate was 92%. For the individual target households (including split-off households as separate) the re-contact rate was […] 90.5%.” We quote here from “The fifth wave of the Indonesia Family Life Survey: Overview and Field Report”, page 8. (Ln 160-162).

**Comment from Reviewer #1**

• Could the author specify which survey weight was used in this study as I expect the survey will have multiple weights. If so, why was that survey weight chosen specifically? (Ln 186)

**Authors’ response**

We used the IFLS 2014 cross-section analysis person weights for the sampling procedures (which oversampled urban areas and some outer provinces) and for attrition (Ln 253-256).

**Comment from Reviewer #1**

• On lines 283-286 the author refers to two correlation analyses which area not sufficiently reported on with CIs and R-values?

**Authors’ response**

We report the detailed results of the logistic regression in the supplementary file, Tables 1-3.

**Comment from Reviewer #2 (Light blue color)**

Is the manuscript technically sound, and do the data support the conclusions?

The manuscript is technically sound. However, the authors should clearly state the community-based health care interventions they are referring to. There is lack of consistence in use of the words in the objective. Is it (factors associated or risk factors or determinants or predictors or relationship) with awareness, treatment and control of hypertension? This should be made consistent in the entire manuscript bearing in mind the study design.

**Authors’ response**

The CBHIs we refer to in this study are “Posbindu PTM and Posyandu Lansia” as we now explain in the introduction, lines 123-124. In the current version of the manuscript, we consistently use the phrase “risk factors” when we explain the risk factors of NCDs and the word “determinants” when we explain the determinants of awareness, treatment, and control of hypertension.

**Comment from Reviewer #2**

"NCD" was already abbreviated in the abstract, hence no need to repeat it in the introduction.

**Authors’ response**

We have removed the repetition of the ‘NCD’’s abbreviation throughout the manuscript.

**Comment from Reviewer #2**

What were the health volunteers trained in? Who trained them and did they follow a curriculum? For how long are they trained? What do the authors mean by modest funding from ministry of health and what does it support? "IFLS" was also already abbreviated and there is no need to repeat it.

**Authors’ response**

Detailed information about the training, trainers and curriculum of Posbindu training as well as funding sources and uses has been added (see introduction Ln 131-147).

**Comment from Reviewer #2**

The lower age cut off for the IFLS-5 participants is not clear (<1).

**Authors’ response**

We have revised <1 to 0 (Ln 159).

**Comment from Reviewer #2**

Let the authors give details of the data that was used. In which wave did these individuals come from? When was this data collected? Did the analysis use the longitudinal data or one survey? What is the study design?

**Authors’ response**

We have provided the information on the details of the data in the methods section in the revised manuscript.

**Comment from Reviewer #2**

The authors should give more details on the Omron self-inflating sphygmomanometers. Who is the manufacturer, in which country, city and when were they last calibrated?

**Authors’ response**

We have provided the information about Omron self-inflating sphygmomanometers, city and country of the manufacturer and date of last calibration (Ln 188-192).

**Comment from Reviewer #2**

The authors should indicate clearly the questions that were included in the survey and were relevant to their study. Was it easy for participants to know if their BP value?

**Authors’ response**

The detailed questions used in the study and coding structure have been added in Supplementary File 1, which will be available online.

**Comment from Reviewer #2**

What power did the study have given the sample size of 30,351 respondents that was used? How were the control variables selected? What was the justification for categorizing each of the variables included in the study?

**Authors’ response**

The 30,351 respondents in ILFS included in this study were representative for 83% of the population aged 18-110 years old in Indonesia. Categorisation of each control variable is presented in the online supplementary file. We follow prior studies in selecting the socio-demographic variables used in this study, as is now explained in the revised manuscript.

**Comment from Reviewer #2**

2. Has the statistical analysis been performed appropriately and rigorously?

The authors do not show the rigor in performing the analysis in systematically and in detail. What do they mean by "Survey weight was used in all analyses to adjust for non-response bias"? Details of what is meant and how it was done should be included.

**Authors’ response**

We used the IFLS-2014 cross-section analysis person weights for the sampling procedures (which oversampled urban areas and some outer provinces) and for attrition. We used the *svy* command in STATA to include the sampling weights in the analysis (Ln 252-254 and Ln 265-267).

**Comment from Reviewer #2**

The word standard deviation (SD) should be included after the word "mean".

**Authors’ response**

We now include the word “standard deviation” after the word “mean”.

**Comment from Reviewer #2**

The outcome (prevalence of hypertension) is common. Was use of OR the best option? Use of OR when the outcome is common over estimates the measure of association (Barros, A.J. and Hirakata, V.N., 2003. Alternatives for logistic regression in cross-sectional studies: an empirical comparison of models that directly estimate the prevalence ratio. BMC medical research methodology, 3(1), p.21)

**Authors’ response**

Following Barros and Hirakata, we have analysed the relationships between participation in the CBHIs for NCDs with awareness, treatment and controlled blood pressure among respondents with hypertension using Poisson regression models. We provide the results in Supplementary Tables 4 to 6.

**Comment from Reviewer #3 (Yellow color)**

Thank you for the opportunity to review this manuscript. Congratulations to the authors for the good piece of work that is largely well written. The study aimed at examining the association of community-based health care interventions with hypertension awareness, treatment and control among Indonesians. The work is based on analysis of a large representative dataset and the employed sound techniques. My major criticism is the presentation and portrayal of this work as one from a longitudinal study, yet the data was from a cross-sectional survey in that the results, discussions and discussions seem overstated. Please see below for my specific comments.

**Authors’ response**

Many thanks for the positive feedback. In the revised manuscript, we clearly state that this study is based on cross-sectional data.

**Comment from Reviewer #3**

Title

The title is misleading and gives an impression of the manuscript being based on a longitudinal evaluation of the community-based health care intervention which was not the case and as cross-sectional data were analysed instead.

Suggested title: Participation in a CBHI and its association with hypertension awareness, control and treatment in Indonesia.

**Authors’ response**

As suggested, we have changed the title to “Participation in CBHIs and its association with hypertension awareness, control and treatment in Indonesia”.

**Comment from Reviewer #3**

Introduction

In addition to stating the gap as whether CBHIs improve awareness, treatment and control, can we highlight the gaps around the association between the variables as a starting point as we not technically studying the intervention effect.

**Authors’ response**

As suggested, we have added some findings and gaps in the literature on whether CBHIs improve awareness, treatment and control in LMIC contexts (Ln 78-115).

**Comment from Reviewer #3**

Line 100: indicate that this is in Indonesia though it still seems overstated.

**Authors’ response**

We deleted this sentence.

**Comment from Reviewer #3**

The aim is correctly stated as examining the association between participation in CBHI and hypertension awareness, treatment and control.

**Authors’ response**

As suggested, we have revised the aim (Ln 145-147).

**Comment from Reviewer #3**

Line 103 to 110 should be omitted. It doesn’t add much information to the study. Only the study aim should be strengthened.

**Authors’ response**

We have omitted the paragraph.

**Comment from Reviewer #3**

Material and methods

Can you move setting to material and methods?

**Authors’ response**

We have removed the setting section and included some information on the setting in the introduction (Ln 116-147).

**Comment from Reviewer #3**

Line 130 should be a continuation with previous paragraph and not stand on its own.

**Authors’ response**

We moved the sentences to introduction.

**Comment from Reviewer #3**

Line 166 ‘on treatment’ not ‘in’

**Authors’ response**

We have revised it (Ln 200).

**Comment from Reviewer #3**

Line 183 How was the question for health insurance phrased or created?

**Authors’ response**

The question to measure having health insurance in the survey is “Are you currently the beneficiary of any type of health insurance?” (Please see Line 230-231.)

**Comment from Reviewer #3**

Provide more details about data collection. What tools were used? was it pretested, validated? how was data collected and managed?

**Authors’ response**

The details of data collection are now explained and include tools, pretesting, validation and data collection and management (Ln 149-186).

**Comment from Reviewer #3**

Results

Line 203 & 204 an omit ‘blood pressure’ after systolic.

**Authors’ response**

The word “blood pressure” after “systolic” has been deleted (Ln 276).

**Comment from Reviewer #3**

Line 207 not clear what is meant by ‘lower’ here.

**Authors’ response**

We have revised the sentence (Ln 279).

**Comment from Reviewer #3**

Line 209 was the proportion 3% or 4%? In line 351, you indicate 4%. Cross check.

**Authors’ response**

Thank you for catching this. It is 4% (Ln 282).

**Comment from Reviewer #3**

Line 214 Can we say respondents instead of sample

**Authors’ response**

We have changed the word “sample” to “respondents” (Ln 286).

**Comment from Reviewer #3**

Table 1 as indicated for means as SE, can we include the same for proportions and include SE. For participation in CBHIs, include the time period of ‘previous 12 months’

**Authors’ response**

As suggested by Reviewer 2, SE should be standard deviation.

**Comment from Reviewer #3**

Line 227 omit ‘a’ primary

**Authors’ response**

The word “a” has been omitted (Ln 299).

**Comment from Reviewer #3**

Line 275 Replace with meaningful title based on what the presented data is about

**Authors’ response**

As suggested, we have revised with a meaningful sub-title (Ln 333-334).

**Comment from Reviewer #3**

Line 276 to 294 use the past tense in describing the results and present results as those from cross sectional data. For example, instead of saying that participation in CBHI is associated with higher awareness, state that having participated in CBHI was associated with …. Or those who had participated had greater odds for awareness, .….. you can also include the 12 months duration since it is not lengthy and helps to contextualise participation. These edits would need to be extended to the discussion section when results are described.

**Authors’ response**

We have made the requested revisions throughout the manuscript.

**Comment from Reviewer #3**

Line 282 replace ‘and’ with ‘or’ and instead of treated respondents, can we have ‘respondents on antihypertensives’?

**Authors’ response**

We have replaced “and” with “or” and “treated respondents” with “respondents on antihypertensives” (Ln 342-343).

**Comment from Reviewer #3**

Line 283 to 286 where is the 99% and 50% coming from? Where is it referenced? If data is not shown for some results, state so

**Authors’ response**

The estimation results were change as we included some control variables as suggested by Reviewer 1 (Ln 340-347).

**Comment from Reviewer #3**

Discussion

Line 297 to 307 Omit paragraph as it is a repetition of the introduction information and doesn’t add much to your study.

**Authors’ response**

We have deleted the paragraph (Ln 364-367).

**Comment from Reviewer #3**

Line 308 to 320 As you make comparisons of hypertension prevalence, can you start with comparisons in Indonesia (312 to 320) before we bring in other countries?

**Authors’ response**

We started with comparisons in Indonesia (Ln 370-378).

**Comment from Reviewer #3**

Line 334 to 349 All comparisons made are based on longitudinal studies which is misleading. It is thus not proper to mention that this study contradicts prior longitudinal studies, yet this was across sectional study and so the comparisons are not similar. It would be better to make appropriate comparisons with cross sectional studies or contextualise the longitudinal ones.

**Authors’ response**

As suggested by Reviewer 1, we have re-written the discussion section so that it directly addresses the aim of the study. Accordingly, we have removed the words “prior longitudinal studies” and changed the comparison so that it is more appropriate.

**Comment from Reviewer #3**

Line 347 Could use ‘may’ instead of ‘will’

**Authors’ response**

We have replaced the word ‘will’ in Line 347 with ‘may’ (Ln 410).

**Comment from Reviewer #3**

Line 351 in addition should be a continuation and not a new paragraph.

**Authors’ response**

We re-organised this sentence (Ln 424).

**Comment from Reviewer #3**

Line 354 Where? In Indonesia?

**Authors’ response**

Yes, we have included the country name (Indonesia) in the sentence on line 354 (Ln 425).

**Comment from Reviewer #3**

Line 361 to 364 talks about the fact that many respondents did not have controlled BP but this is not a study limitation.

**Authors’ response**

We have omitted the sentence in lines 361 to 364 (Ln 430-438).

**Comment from Reviewer #3**

Line 364 to 365 Absence of medication data doesn’t seem to fit our study objectives and not carrying out further investigations does not affect our study to state it as a limitation.

**Authors’ response**

We have removed the absence of medication as one of the limitations of the study (Ln 430-438).

**Comment from Reviewer #3**

Line 365 How would not capturing non-pharmacological treatment strategies lead to an underestimation of treatment rates? It could have led to an overestimation of the CBHI effect which limitation should be highlighted.

**Authors’ response**

We have revised the sentence in Line 433-435. It now reads:

“In addition, this study did not capture non-pharmacological treatment strategies such as modifications in diet and physical activity, which may have yielded overestimation of the CBHI effect on the reduction of hypertension rates.”

**Comment from Reviewer #3**

The fact that the participation of respondents could not been quantified is also a study limitation and thus it is not clear whether there was a relationship with duration, frequency or mode of participation with the outcomes. Moreover, the participation rate was very low (4%).

**Authors’ response**

We have included the absence of the information regarding the duration, frequency and mode of participation to the CBHI and the low proportion of the respondents attending CBHIs in the study limitations (Ln 430-438).

**Comment from Reviewer #3**

Line 373 The stated contributions are overstated, and some are not in context with the study findings. Revise these

**Authors’ response**

We have revised the sentence on line 373. It now reads:

“Despite these limitations, the present study makes several noteworthy contributions to the literature on CBHIs and NCD prevention as well as assisting policy makers in determining the role of CBHIs in increasing hypertension awareness and treatment in Indonesia.” (Ln 441-452)

**Comment from Reviewer #3**

Abstract

Conclusion is misleading. The CBHI described does not include lifestyle modification on which the conclusion is based.

**Authors’ response**

We have revised the conclusion in the abstract. It now reads:

“Our data emphasise the importance of CBHIs for NCDs to improve the awareness and treatment of hypertension in the Indonesian population.” (Ln 46-47)

**Comment from Reviewer #3**

General

The writing is laden with repetitions, inconsistencies in grammar and is not succinct. A through grammatical check will help improve it. For example, repetitions around ‘the respondents’ after it has already been mentioned.

**Authors’ response**

Thank you for raising this point. We have asked a native speaker and professional editor to perform a full and comprehensive editing of our manuscript in order to avoid grammatical errors in the revised manuscript.

**Comment from Reviewer #3**

References

Reference 3 Edit world Health organization as author.

**Authors’ response**

We have revised Reference 3 into “World Health Organization” (Ln 459).
